# Phase contrast time-lapse microscopy datasets with automated and manual cell tracking annotations

**DOI:** 10.1038/sdata.2018.237

**Published:** 2018-11-13

**Authors:** Dai Fei Elmer Ker, Sungeun Eom, Sho Sanami, Ryoma Bise, Corinne Pascale, Zhaozheng Yin, Seung-il Huh, Elvira Osuna-Highley, Silvina N. Junkers, Casey J. Helfrich, Peter Yongwen Liang, Jiyan Pan, Soojin Jeong, Steven S. Kang, Jinyu Liu, Ritchie Nicholson, Michael F. Sandbothe, Phu T. Van, Anan Liu, Mei Chen, Takeo Kanade, Lee E. Weiss, Phil G. Campbell

**Affiliations:** 1 Department of Biological Sciences, Carnegie Mellon University, Pittsburgh, USA; 2 Institute for Tissue Engineering and Regenerative Medicine, The Chinese University of Hong Kong, Shatin, Hong Kong SAR; 3 School of Biomedical Sciences, Faculty of Medicine, The Chinese University of Hong Kong, Shatin, Hong Kong SAR; 4 Robotics Institute, Carnegie Mellon University, Pittsburgh, USA; 5 Dai Nippon Printing, Tokyo, Japan; 6 Department of Advanced Information Technology, Kyushu University, Fukuoka, Japan; 7 Engineering Research Accelerator, Carnegie Mellon University, Pittsburgh, USA; 8 Department of Computer Science, Missouri University of Science and Technology, Rolla, USA; 9 Intel Labs Pittsburgh, Pittsburgh, USA; 10 Department of Computer Science, Carnegie Mellon University, Pittsburgh, USA; 11 School of Electrical and Information Engineering, Tianjin University, Tianjin, China; 12 Department of Electrical and Computer Engineering, University at Albany, State University of New York, Albany, USA; 13 Department of Biomedical Engineering, Carnegie Mellon University, Pittsburgh, USA

**Keywords:** Computational biology and bioinformatics, Phase-contrast microscopy

## Abstract

Phase contrast time-lapse microscopy is a non-destructive technique that generates large volumes of image-based information to quantify the behaviour of individual cells or cell populations. To guide the development of algorithms for computer-aided cell tracking and analysis, 48 time-lapse image sequences, each spanning approximately 3.5 days, were generated with accompanying ground truths for C2C12 myoblast cells cultured under 4 different media conditions, including with fibroblast growth factor 2 (FGF2), bone morphogenetic protein 2 (BMP2), FGF2 + BMP2, and control (no growth factor). The ground truths generated contain information for tracking at least 3 parent cells and their descendants within these datasets and were validated using a two-tier system of manual curation. This comprehensive, validated dataset will be useful in advancing the development of computer-aided cell tracking algorithms and function as a benchmark, providing an invaluable opportunity to deepen our understanding of individual and population-based cell dynamics for biomedical research.

## Background & Summary

Studying the dynamic behaviour of cells and their interactions with the local microenvironment requires large datasets and accurate ground truths to develop sophisticated quantification tools. While advances in microscopy automation and computational hardware have simplified the acquisition of live cell imaging data, many biological insights remain undiscovered and ‘buried’ by the sheer data volume and intractable nature of analysis. As such, accurate computer algorithms for precise cell tracking are crucial towards uncovering new biological phenomenon. For example, the ‘Baxter’ algorithm was used to track cells and construct lineage relationships to determine the effects of substrate stiffness on muscle stem cell behaviour^1^. The results show that this algorithm not only reduced analysis time by approximately 90%, with a low 1% error rate, but also demonstrated that pliant hydrogels promoted muscle stem cell potency by producing twice as many clones to increase cell survival^1^. In contrast, muscle stem cells cultured on rigid hydrogels did not exhibit any changes in overall cell numbers due to similar rates of cell division and cell death^1^. Such success has also led to the availability of commercial cell tracking software^2,3^. Together, both biology and the biomedical sciences stand to benefit from the development of accurate cell tracking algorithms.

Despite their reported success, a crucial bottleneck still remains in the universal applicability of cell tracking algorithms, especially under drastically different experimental conditions. At minimum, cell tracking algorithms comprise at least 2 modules – object (cell) detection and tracking. The method of implementing these modules is heavily reliant on the particular experimental conditions, which presents different challenges and requires customized solutions. For example, the experimental conditions of wide field-of-view fluorescence imaging are such that individual cells are reduced to small pixel dimensions relative to the overall image size against a noisy background^4^. A feedback loop between tracking and detection modules was necessary to reject more than 97% of false positives in terms of cell identification. Where such customized solutions are not possible, as with the case of commercially available software, accuracy becomes a crucial outcome that must be properly monitored and taken into account. For example, attempts were made to discard errors during automated tracking of T lymphocytes by deleting cell tracks that were less than 60 s in duration^5^. Such attempts, however, do not account for tracking errors that persist longer than this arbitrary cut-off threshold. This is critical since a tracking error made early on can propagate into additional errors throughout the remainder of the image sequence, confounding biological interpretation of the results. Cell tracking algorithms must exhibit robust performance under diverse experimental conditions to improve their universal applicability.

In order to improve the universal applicability of cell tracking algorithms, diverse datasets and ground truths are needed. In this study, mouse C2C12 muscle progenitor cells were cultured under 4 different media conditions for approximately 3.5 days to generate 48 phase-contrast time-lapse image sequences with accompanying ground truths for 3 parent cells (approximately 10% of starting cell population). Phase contrast microscopy was chosen because this imaging modality is highly prevalent and non-destructive (i.e. no phototoxicity), allowing long-term imaging of rapid population growth. Four different culture conditions, including media with fibroblast growth factor2 (FGF2), bone morphogenetic protein 2 (BMP2), FGF2 + BMP2, and control (no growth factor), were chosen since they dramatically alter C2C12 cell morphology and will be useful for developing robust cell tracking algorithms. To facilitate appropriate annotation of this dataset, we have also developed a data framework to assign tracking-relevant states to cells, including but not limited to mitosis, apoptosis, and entering or departing a field-of-view. Together, this large image dataset and our proposed data framework has high reusability and will be vital for assessing the accuracy of computer-aided cell tracking algorithms under robust conditions, thus advancing the development of universal automated cell tracking systems.

## Methods

### Cell culture

Mouse C2C12 cells (ATTC, Manassas, VA) were grown in Dulbecco’s Modified Eagle’s Media (DMEM; Invitrogen, Carlsbad, CA), 10% fetal bovine serum (Invitrogen, Carlsbad, CA) and 1% penicillin-streptomycin (PS; Invitrogen, Carlsbad, CA). Cells were kept at 37 ^o^C, 5% CO_2_ in a humidified incubator.

In this study, C2C12 cells were seeded at a density of 2 × 10^4^ cells per 35 mm Petri dish for approximately 3 to 6 h. The media was subsequently changed and cells were grown under 4 different conditions: 1) Control (Untreated), 2) 100 ng/mL FGF2 (Peptrotech Inc., Rocky Hill, NJ), 3) 100 ng/mL BMP2 (Medtronic, Minneapolis, MN) and 4) 100 ng/mL FGF2 and 100 ng/mL BMP2 in complete media over the course of approximately 3.5 days.

### Phase-contrast time-lapse microscopy

Time-lapse phase-contrast microscopy was performed using a Zeiss Axiovert T135V microscope (Carl Zeiss Microimaging, Thornwood, NY) equipped with a 5X, 0.15 N.A. phase-contrast objective, a custom-stage incubator capable of housing up to four 35 mm Petri dishes, and *In Vitro* software 3.2 (Media Cybernetics Inc., Bethesda, MD). Four fields of view representative of the cell density from four dishes, each with different treatments (FGF2, BMP2, FGF2 + BMP2, and control), were selected, resulting in a total of 16 fields of view per experiment. Each experiment was repeated a total of three times, resulting in a total of 48 image sequences (12 phase-contrast time-lapse microscopy image sequences per treatment group). Images were acquired at a frequency of every 5 minutes over a course of 3.5 days and each image sequence contained approximately 1013 to 1062 frames. Microscope images were 1392 × 1040 pixels with a resolution of 1.3 μm/pixel. The organization of the datasets is listed in Table 1.

### Cell tracking annotation states

Cell tracking results from automated computer-aided cell tracking and those manually generated by human experts utilized the same annotation scheme, which contained built-in contingencies for reflecting uncertainty during assignment of cell states as well as difficulty in tracking single cells versus groups of cells. To facilitate tracking-related cell annotations, it was necessary to create a data framework, which is listed in Table 2. This data framework allowed cells to be labelled with a total of 18 states that are listed in Table 3. These include ‘Invalid’, ‘New’, ‘Newborn’, ‘Divided’, ‘Normal’, ‘Apoptotic/Mitotic’, ‘Apoptotic’, ‘Mitotic’, ‘Maybe Dead’, ‘Dead’, ‘Fused’, ‘Departed’, ‘Entered’, ‘Appeared’, ‘New Group’, ‘Grouped’, ‘Differentiated’, ‘Maybe Lost’, and ‘Lost’.

### Manually-generated cell tracking ground truth annotation by human experts

The microscope image data obtained were manually annotated using our in-house developed software (unpublished). Cells were individually tagged by placing a marker at the center of the cell (cell centroid) at approximately every 1 to 8 frames with this software, which then applied interpolation to determine the cell centroid between these frames. At the appropriate frame, individual cells were also assigned a label to highlight the status of the cell. Due to the time-consuming process of manual annotation, a minimum of 3 cells per image sequence representing approximately 10% of the initial number of cells in the field-of-view were manually annotated from the beginning of the image sequence through to the end, resulting in 48 partially-annotated image sequences. Also, a single image sequence (100 ng/mL BMP2, Dataset 01) was manually annotated for all cells for 780 frames, representing 65 h. Ground truths for each image sequence were produced by trained personnel and subsequently curated by expert annotators with at least five years of experience with mammalian cell culture. The time required to annotate a single cell and all its descendants was approximately 2–3 h. For an image sequence consisting of approximately 30 cells, this would require 60–90 h per image sequence or 2,880–4,320 h for 48 image sequences. Although the time required to partially annotate 48 image sequences is estimated to be approximately 288–432 h (3 cells × 2–3 h per cell × 48 image sequences), it took 1.5 years to complete the annotations due to logistics and personnel training. The XML Schema of the resulting tracking file was validated using XML Explorer 4.0.5.0 (https://xmlexplorer.codeplex.com/).

### Computer-aided cell tracking annotation

Computer-based cell tracking annotations were obtained using our in-house developed tracking system. This system employs a tracking-by-detection approach, which first segments cells and then associates those cells over consecutive frames. The tracking algorithm consists of three modules – (1) segmentation^6^, (2) mitosis detection^7^, and (3) association^8–10^. Similar to human-generated ground truths, the automated cell tracking system marks the cell centroid and labels the cell status but does so for all cells in the image sequence. The XML Schema of the resulting tracking file was validated using XML Explorer 4.0.5.0 (https://xmlexplorer.codeplex.com/). These automated cell tracking results (circa 2011) are provided to facilitate comparison with new tracking algorithms.

During segmentation, cells are segmented from background using a microscope image restoration process^6^. Based on a microscopy imaging model^6^, the process removes artifacts in phase-contrast microscopy, such as the halo and shading effects, and restore the artifact-free images in which background pixels have uniform zero values and foreground pixels positive values. On these restored high-contrast images, a simple thresholding method was sufficient to separate a group of cell positive pixels (termed ‘blobs’) from background pixels^6^.

For mitosis detection, images were processed using a three-step approach to identify the end of cytokinesis^7^, facilitating improved tracking performance by establishing accurate parent-daughter relationships. The three-step approach consisted of – (1) Candidate patch sequence construction, (2) Feature extraction, and (3) Identification of mitosis and localization of this event. Briefly, during the first step, potential regions that may contain mitotic events were located in the image sequence based on their pixel intensity and these areas were subsequently cropped to construct small-size candidate patch sequences. This step narrowed down the available search space required for locating mitotic cells to facilitate efficient mitosis detection and spatially locate this cell division event. In the second step, visual features (a set of numbers that describe the characteristics of an image patch) were extracted from each candidate patch. Since cell size does not vary significantly during cell division and each cell can freely rotate when dividing, these visual features were extracted with a unique scale and a rotation invariance scheme was applied. In the third step, the visual features were examined by a probabilistic model constructed using machine learning approaches^7^ to determine whether each candidate patch sequence contained a cell division event and if so, detect the temporal locations of the cell division event in the sequence.

So far, the segmentation algorithm identified blobs from the original input images that contained either individual cells or multiple cells clustered together, and the mitosis detection algorithm determined when and where a cell blob completed cytokinesis and divided into two cell blobs. The association step now aimed for correlating and linking cell identities along the whole image sequence for cell tracking. Based on the outputs of the segmentation and mitosis detection modules, hypotheses were first constructed about a link or correspondence of the identities of segmented individual cells (or blobs) between two successive frames (frame t = n and frame t = n + 1), which are termed ‘cell tracks’^8–10^. The hypothesis generation also accounted for scenarios that include cell migration within the field-of-view, cell migration into and out of the field-of-view, cell division, and cell clustering. Then, from the entire hypothesis set the most probable sequences of links or correspondences were solved using linear programming^8–10^. This resulted in the linkage of all individual cell tracks together with their characteristics (position, area, shape, etc.).

### Code availability

The code for the in-house developed system that produced human-generated ground truths and computer-aided annotations is not available due to our lack of a commercial license for distribution. However, we have provided R computer code (Data Citation 1) in an Adobe Acrobat PDF file to facilitate opening, visualization, and analysis of image and annotation files. The FiJi distribution of ImageJ 1.51n (https://fiji.sc/)^11,12^ was used to compile image sequences and perform image verification. XML Explorer 4.0.5.0 (https://xmlexplorer.codeplex.com/) was used to perform XML Schema validation of human-generated ground truths and computer-aided cell annotations.

## Data records

**Figure 1:**
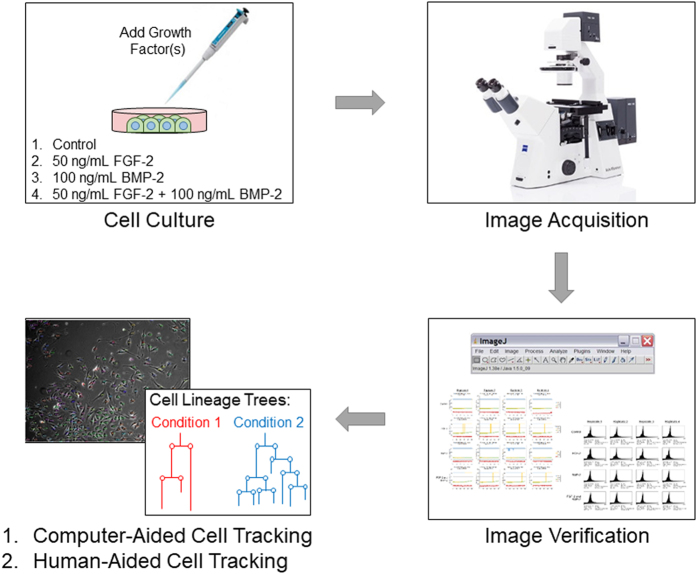
Overall experimental workflow for generating phase-contrast time-lapse image data, image verification as well as computer-aided and manually-generated cell tracking annotations. Three phase-contrast time-lapse microscopy image datasets were obtained independently. Each dataset consisting of four culture conditions – Control (growth media), 50 ng/mL FGF2, 100 ng/mL BMP2, and 50 ng/mL FGF2 + 100 ng/mL BMP2. Four replicates were imaged per dataset at a frequency of 5 minute intervals over approximately 3.5 days. Each image sequence was compiled and verified using the FiJi distribution of ImageJ 1.51n^11,12^. Computer-aided cell tracking annotations were produced using our previously published cell tracking system which consists of segmentation^6^, mitosis detection^7^, and association^8–10^ modules. Ground truth annotations were produced by trained personnel and subsequently curated by another annotator with at least five years of experience with mammalian cell culture. Such data can be used to generate cell lineage trees.

The image and annotation data (Data Citation 1) consist of the following:

1. 49,919 phase-contrast microscope images (TIFF format, 1392 × 1040 pixels, 16 Bit depth) organized into 3 datasets comprising of 48 image sequences. There are a total of four culture conditions – Control (no growth factor), 50 ng/mL FGF2, 100 ng/mL BMP2, and 50 ng/mL FGF2 + 100 ng/mL BMP2 with 12 image sequences for each culture condition (Table 1 and Fig. 1).
2. 48 worksheet files (CSV format) detailing pixel intensity attributes of each image sequence and used for validating individual images (Table 1).
3. 48 manual cell tracking annotations (XML format, Tables 1, 2 and 3) generated from a group of trained personnel and additionally curated by expert annotators with at least five years of cell culture experience.
4. 48 computer-aided cell tracking annotations (XML format, Tables 1, 2 and 3) generated from our previously published algorithms (circa 2011) which utilize three modules for segmentation^6^, mitosis detection^7^, and (3) track association^8–10^.
5. 1 XML Schema file (XSD format, Tables 1, 2 and 3) for validating cell tracking annotation XML files (Table 1). This file also specifies the structure of the data framework for both human-generated ground truths and computer-aided cell tracking annotations (Table 2) including 19 possible annotations of cell status and parameters for visualizing cell tracking results (Table 3).
6. 1 Adobe Acrobat file (PDF format) containing R computer code for opening, visualization, and analysis of image and annotation files. Image-wise, the computer code enables visualization of image sequences and tracking annotations together or separately as well as re-sizing of image files. Annotation-wise, the computer code enables importing of computer- or human-annotated data as R data objects for subsequent biological analysis such as plotting of cell lineage trees. Detailed instructions on usage are included in the ‘Description and Instructions for Use’ section of the file.

The image, annotation, and data validation files are divided into 3 folders, representing 3 independent experiments. The organization of the data is detailed in Table 1 while the proposed data framework for cell tracking annotation is detailed in Tables 2 and 3.

## Technical Validation

### Image verification

**Figure 2:**
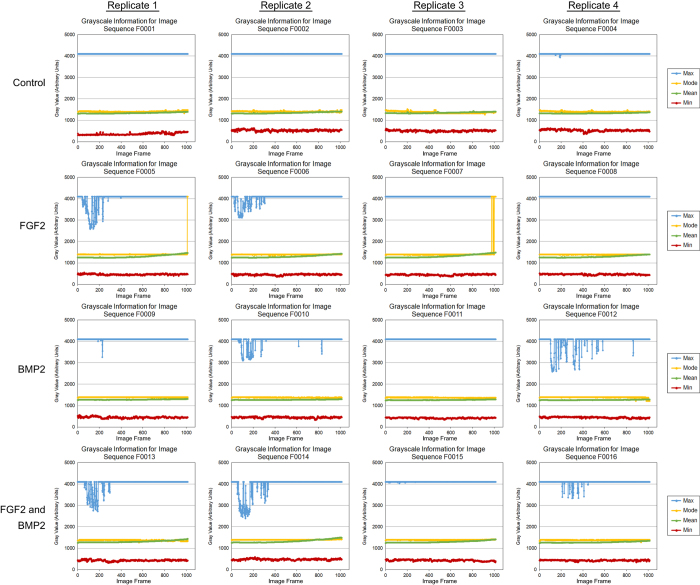
Individual verification of images for dataset 01 (090303-C2C12P15-FGF2,BMP2). The minimum, maximum, modal, and mean pixel values (arbitrary units) for each individual image of the sequence was plotted as a function of image frame number. The mean, minimum, and maximum pixel intensity remained relatively unchanged throughout the image sequence. Addition of FGF2 resulted in cell morphology changes that increased pixel intensity. Each dataset consists of four culture conditions – Control (growth media), 50 ng/mL FGF2, 100 ng/mL BMP2, and 50 ng/mL FGF2 + 100 ng/mL BMP2. Four replicates were imaged per dataset at a frequency of 5 minute intervals over approximately 84.4 h.

**Figure 3:**
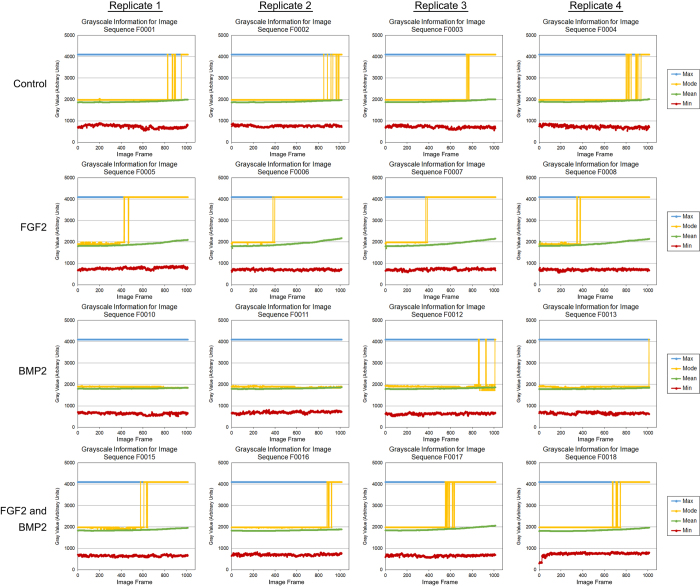
Individual verification of images for dataset 02 (090318-C2C12P7-FGF2,BMP2). The minimum, maximum, modal, and mean pixel values (arbitrary units) for each individual image of the sequence was plotted as a function of image frame number. The mean, minimum, and maximum pixel intensity remained relatively unchanged throughout the image sequence. Addition of FGF2 resulted in cell morphology changes that increased pixel intensity. Each dataset consists of four culture conditions – Control (growth media), 50 ng/mL FGF2, 100 ng/mL BMP2, and 50 ng/mL FGF2 + 100 ng/mL BMP2. Four replicates were imaged per dataset at a frequency of 5 minute intervals over approximately 88.6 h.

**Figure 4:**
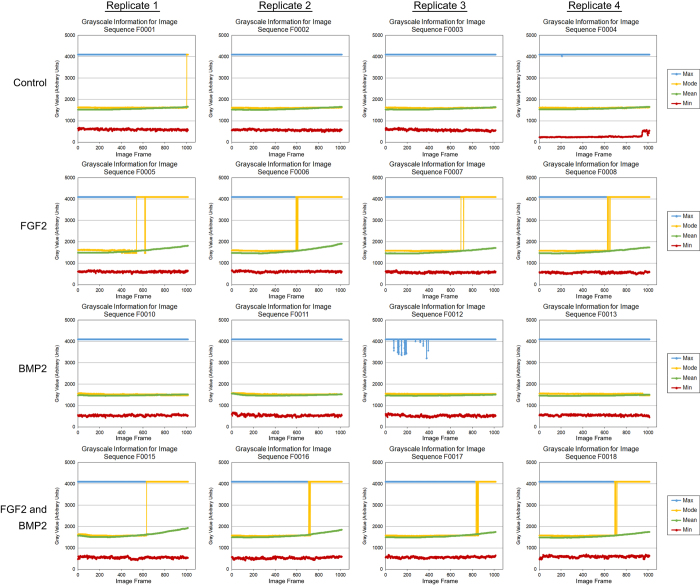
Individual verification of images for dataset 03 (090325-C2C12P12-FGF2,BMP2). The minimum, maximum, modal, and mean pixel values (arbitrary units) for each individual image of the sequence was plotted as a function of image frame number. The mean, minimum, and maximum pixel intensity remained relatively unchanged throughout the image sequence. Addition of FGF2 resulted in cell morphology changes that increased pixel intensity. Each dataset consists of four culture conditions – Control (growth media), 50 ng/mL FGF2, 100 ng/mL BMP2, and 50 ng/mL FGF2 + 100 ng/mL BMP2. Four replicates were imaged per dataset at a frequency of 5 minute intervals over approximately 87.0 h.

**Figure 5:**
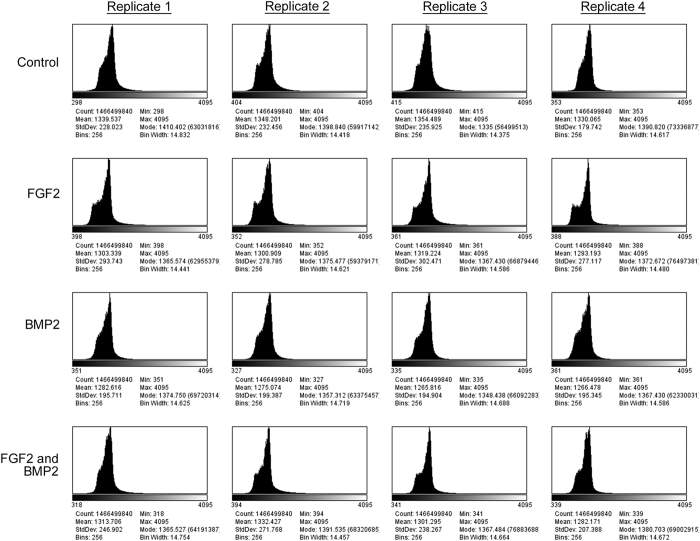
Overall verification of image sequence for dataset 01 (090303-C2C12P15-FGF2,BMP2). A histogram showing the range of pixel values for the entire image sequence was plotted. The total pixel count, minimum pixel value, maximum pixel value, modal pixel value, mean pixel value, standard deviations, and histogram bin parameters are shown. Each dataset consists of four culture conditions – Control (growth media), 50 ng/mL FGF2, 100 ng/mL BMP2, and 50 ng/mL FGF2 + 100 ng/mL BMP2. Four replicates were imaged per dataset at a frequency of 5 min intervals over approximately 84.4 h.

**Figure 6:**
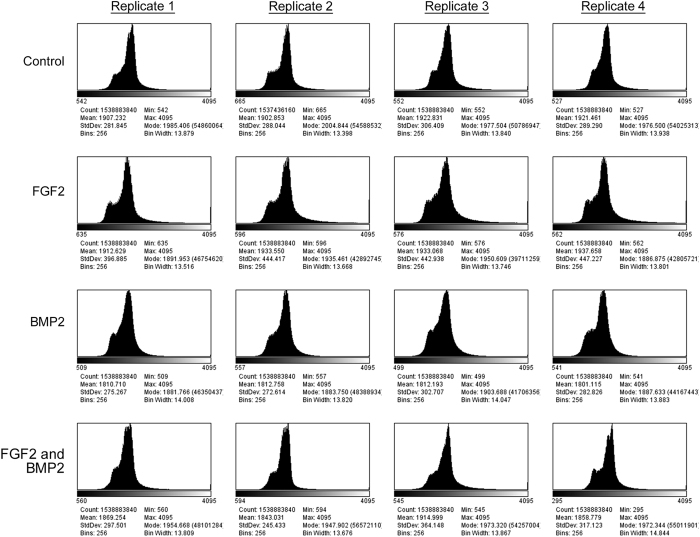
Overall verification of image sequence for dataset 02 (090318-C2C12P7-FGF2,BMP2). A histogram showing the range of pixel values for the entire image sequence was plotted. The total pixel count, minimum pixel value, maximum pixel value, modal pixel value, mean pixel value, standard deviations, and histogram bin parameters are shown. Each dataset consists of four culture conditions – Control (growth media), 50 ng/mL FGF2, 100 ng/mL BMP2, and 50 ng/mL FGF2 + 100 ng/mL BMP2. Four replicates were imaged per dataset at a frequency of 5 min intervals over approximately 88.6 h.

**Figure 7:**
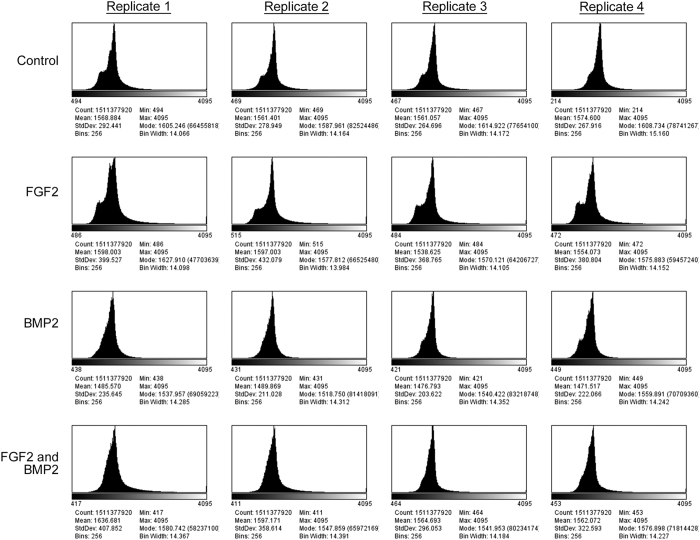
Overall verification of image sequence for dataset 03 (090325-C2C12P12-FGF2,BMP2). A histogram showing the range of pixel values for the entire image sequence was plotted. The total pixel count, minimum pixel value, maximum pixel value, modal pixel value, mean pixel value, standard deviations, and histogram bin parameters are shown. Each dataset consists of four culture conditions – Control (growth media), 50 ng/mL FGF2, 100 ng/mL BMP2, and 50 ng/mL FGF2 + 100 ng/mL BMP2. Four replicates were imaged per dataset at a frequency of 5 min intervals over approximately 87.0 h.

To determine the quality of individual images, the minimum, maximum, mean and modal pixel values were measured using the FiJi distribution of ImageJ 1.51n (https://fiji.sc/)^11,12^ and plotted as a function of image frame number (Figs 2, 3, 4). To determine the overall quality of an image sequence, a histogram of pixel values was also plotted (Figs 5, 6, 7). In this study, images had minimum pixel values that ranged from 298 to 665 and a maximum pixel value of 4095, while the overall mean pixel values of image sequences ranged from 1282 to 1937, indicating high dynamic range (Figs 2, 3, 4, 5, 6, 7).

### Cell tracking annotation verification

Ground truths for manual cell tracking annotations were produced by trained personnel and subsequently curated by expert annotators with more than five years of cell culture experience. Also, XML Explorer 4.0.5.0 (https://xmlexplorer.codeplex.com/) was used to validate the XML Schema of human-generated ground truths and computer-aided cell annotations – no errors were detected.

### Usage notes

Most aspects related to use of the data set are self-explanatory. The phase-contrast time-lapse microscopy and cell tracking annotation data may be opened using appropriate imaging and XML software, respectively. However, based on our experience, we recommend using software that couples imaging and data analysis functionality together. This includes but is not limited to ImageJ (https://fiji.sc/)^11,12^, R/RStudio (https://cran.r-project.org/ and https://www.rstudio.com/) and MATLAB (https://www.mathworks.com/) software. We have also provided a PDF document that includes R computer code to facilitate reading, visualization and analysis of this data. Detailed instructions on code usage are included in the introductory section of the PDF document.

## Additional information

**How to cite this article**: Ker, D. F. E. *et al.* Phase contrast time-lapse microscopy datasets with automated and manual cell tracking annotations. *Sci. Data*. 5:180237 doi: 10.1038/sdata.2018.237 (2018).

**Publisher’s note**: Springer Nature remains neutral with regard to jurisdictional claims in published maps and institutional affiliations.

## Supplementary Material



## Figures and Tables

**Table 1:** Dataset information and data folder names.

**Groups**	**Dataset 01**	**Dataset 02**	**Dataset 03**
Dataset Location	**Data Citation 1**		
Contains	3 folders containing 3 datasets		
Date of Experiment	3^rd^ March 2009	18^th^ March 2009	25^th^ March 2009
Dataset Folder Name	090303-C2C12P15-FGF2,BMP2	090318-C2C12P7-FGF2,BMP2	090325-C2C12P12-FGF2,BMP2
Subfolder Names For Control Experimental Condition	exp1_F0001 Dataexp1_F0002 Dataexp1_F0003 Dataexp1_F0004 Data	exp1_F0001 Dataexp1_F0002 Dataexp1_F0003 Dataexp1_F0004 Data	exp1_F0001 Dataexp1_F0002 Dataexp1_F0003 Dataexp1_F0004 Data
Subfolder Names For 50 ng/mL FGF2 Experimental Condition	exp1_F0005 Dataexp1_F0006 Dataexp1_F0007 Dataexp1_F0008 Data	exp1_F0005 Dataexp1_F0006 Dataexp1_F0007 Dataexp1_F0008 Data	exp1_F0005 Dataexp1_F0006 Dataexp1_F0007 Dataexp1_F0008 Data
Subfolder Names For 100 ng/mL BMP2 Experimental Condition	exp1_F0009 Dataexp1_F0010 Dataexp1_F0011 Dataexp1_F0012 Data	exp1_F0010 Dataexp1_F0011 Dataexp1_F0012 Dataexp1_F0013 Data	exp1_F0010 Dataexp1_F0011 Dataexp1_F0012 Dataexp1_F0013 Data
Subfolder Names for 50 ng/mL FGF2 + 100 ng/mL BMP2 Experimental Condition	exp1_F0013 Dataexp1_F0014 Dataexp1_F0015 Dataexp1_F0016 Data	exp1_F0015 Dataexp1_F0016 Dataexp1_F0017 Dataexp1_F0018 Data	exp1_F0015 Dataexp1_F0016 Dataexp1_F0017 Dataexp1_F0018 Data
Subfolder Names for Human-Generated Ground Truths	Annotation_Human	Annotation_Human	Annotation_Human
Subfolder Names for Computer-Aided Cell Annotation	Annotation_Computer	Annotation_Computer	Annotation_Computer
Subfolder Names for Data Verification	Image_Verification	Image_Verification	Image_Verification
Filename for XML Schema Verification of Cell Tracking Annotations	CellTrackingAnnotationSchema.xsd (located in the root directory of Data Citation 1)		

**Table 2:** Structure of annotation data, attributes, and attribute description.

**No**	**<Element>(Description)**	**Contains**	**Attributes **	**Attribute Description**	**Usage**
**1.**	<AnnotationDocument> (root element)	This element encapsulates all other elements.	Version={[0.0,inf]}DateTime={“ ”}Host={“ ”}	Specifies the version of the annotation data framework.Specifies the date and time.Specifies the host.	AnnotationDocument <AnnotationDocument Version = “”>
**2.**	<fs> (folder structure; houses multiple sets of tracking annotations using <f> elements)	This element contains <f> elements.	None	N/A.	AnnotationDocument / fs<fs><f>…</f><f> … </f></fs>
**3.**	<f> (folder; describes visualization parameters for a set of tracking annotations, houses a set of tracking annotations using <as> elements)	This element contains a <as> element	name={“ ”}vis={0|1}	Specifies the name of the folder.^0^ Hides the folder.^1^ Displays the folder.	AnnotationDocument / fs / f<f name=“” vis=“”><as> … </as></f>
**4.**	<as> (annotation structure; houses annotations associated with cells at the beginning of the image sequence and/or their descendant cells in <a> elements)	This element contains a number of <a> elements	None	N/A.	AnnotationDocument / fs / f / as <as> <a> … </a> <a> … </a> </as>
**5.**	<a> (annotation; describes visualization parameters for a cell, houses annotations associated with the cell in the <ss> element and its descendant cells in <as> elements)	This element contains one <ss> and a number of <as> child elements	id={[1.inf]}type={“ ”}vis={0|1}brush={“#000000”}pen={“#000000”}	Specifies the cell’s unique ID numberSpecifies annotation type.^0^ Hides the folder.^1^ Displays the folder.Specifies the brush (fill) color.Specifies the pen (border) color.	AnnotationDocument / fs / f / as / a <a id = “” type = “” vis=“” brush=“” pen=“”> <ss> … </ss> <as> … </as> <as> … </as></a>
**6a.**	<ss> (state structure; describes the first frame a cell appears, houses all annotations associated with the status of the cell in <s> elements)	This element contains a number of <s> elements	fi={[0, inf]}	Specifies the first frame index that the cell appears.	AnnotationDocument / fs / f / as / a / ss<ss fi=“”> <s> … </s><s> … </s></ss>
**6b.**	<s> (state; describes the status of a cell at a particular frame index)	This element describes the state of the cell in the frame index	i={[0,inf]}x={[0.0,inf]}y={[0.0,inf]}f={“ ”}s={[0,18]}	Specifies the frame index.Specifies the cell’s x-position in the image.Specifies the cell’s y-position in the image.Specifies whether the result was generated from interpolation or “I” (for human-generated ground truths).Specifies cell state (Table 3).	AnnotationDocument / fs / f / as / a / ss / s <s i=“” x=“” y=“” f=“” s=“”> </s>

**Table 3:** Possible states for cell annotation.

**Status Number**	**Status Name**	**Status Description**	**Recommended Usage**
0	Invalid	Invalid cell status	This state may be applied to indicate error.
1	New	The cell appears for the first time in the image sequence.	This state may be applied to cells with normal status in the very first frame of the image sequence.
2	Newborn	The cell is a newborn (resulting from cell division).	This state may be applied to the first frame when newborn daughter cells have appeared as a result of cell division.
3	Divided	The cell divided.	This state may be applied to the last frame when mitotic cells have divided.
4	Normal	The cell does not have any particular event associated with it.	This state may be applied for the frame duration when the status of the cell cannot be determined by any other status code.
5	Apoptotic/Mitotic	The cell is either apoptotic or mitotic.	This state may be applied for the frame duration when its exact status with regards to apoptosis and mitosis cannot be determined (due to similarities in the morphological appearance of cells during apoptosis and mitosis).
6	Apoptotic	The cell is apoptotic.	This state may be applied for the frame duration when the cell is undergoing apoptosis.
7	Mitotic	The cell is mitotic.	This state may be applied for the frame duration when the cell is undergoing mitosis.
8	Maybe Dead	The cell maybe dead.	This state may be applied for the frame duration when its exact status with regards to cell death cannot be determined. This state reflects uncertainty in cell trajectories.
9	Dead	The cell is dead.	This state may be applied to the last frame of a cell undergoing apoptosis and/or for the frame duration the dead cell debris persists in the image sequence.
10	Fused	The cells adjacent to each/one another have fused and are now indistinguishable.	This state may be applied to the last frame of a cell prior to fusing as well as for the remaining frame duration of its fusing partner cell. The fusing partner cell’s trajectory will continue being tracked until its cell status changes (e.g. the cell undergoes mitosis, departs the field-of-view, etc).
11	Departed	The cell left the field-of-view.	This state may be applied to the last frame of a cell prior to departing the field-of-view.
12	Entered	The cell entered the field-of-view.	This state may be applied to the first frame of a cell as it enters the field-of-view.
13	Appeared	A cell has appeared.	This state may be applied to the first frame when a new cell whose appearance cannot be explained by mitosis or a cell entering the field-of-view. This state reflects uncertainty in cell trajectories.
14	New Group	The cell joins or has formed a group.	This state may be applied the first frame when two or more cells can no longer be tracked as single cells and are instead tracked as a group. This state reflects difficulty in assigning cell trajectories.
15	Grouped	The cell is in a group.	This state may be applied for the remaining frame duration when two or more cells can no longer be tracked as single cells and are instead tracked as a group. This state reflects difficulty in assigning cell trajectories.
16	Differentiated From the Group	The cell has left the group.	This state may be applied to the first frame when a cell has left a group of cells. This state reflects difficulty in assigning cell trajectories.
17	Maybe Lost	The cell maybe lost.	This state may be applied for the frame duration when a cell’s trajectory is tentative and uncertain. This state reflects uncertainty in cell trajectories.
18	Lost	The cell has been lost.	This state may be applied to the last frame when a cell’s trajectory can still be tracked. This state reflects difficulty in assigning cell trajectories.
